# P55 Antimicrobial stewardship education for pre-registration medical and healthcare students: a systematic review

**DOI:** 10.1093/jacamr/dlaf230.062

**Published:** 2025-12-04

**Authors:** Simonne Weeks, Aaron Drovandi, Frances Garraghan, Robert Shorten, Rebecca Turner, Lucie Byrne-Davis, Joanne Hart

**Affiliations:** School of Medical Sciences, Faculty of Biology, Medicine and Health, University of Manchester, Manchester, UK; British Society for Antimicrobial Chemotherapy, Birmingham, UK; School of Medical Sciences, Faculty of Biology, Medicine and Health, University of Manchester, Manchester, UK; British Society for Antimicrobial Chemotherapy, Birmingham, UK; Lancashire Teaching Hospitals NHS Foundation Trust, UK; School of Medical Sciences, Faculty of Biology, Medicine and Health, University of Manchester, Manchester, UK; School of Medical Sciences, Faculty of Biology, Medicine and Health, University of Manchester, Manchester, UK; School of Medical Sciences, Faculty of Biology, Medicine and Health, University of Manchester, Manchester, UK

## Abstract

**Background:**

Antimicrobial resistance (AMR) is a leading global health challenge responsible for over 1.27 million deaths annually and rising clinical and economic burdens on healthcare systems. Inappropriate prescribing is the primary driver of resistance, and preparing future healthcare professionals to prescribe responsibly is important. Antimicrobial stewardship (AMS) provides a structured approach to optimizing antimicrobial use and is recognized by the WHO as a priority. Undergraduate training offers a critical opportunity to embed stewardship principles before graduates assume independent roles. Despite this, AMS education remains inconsistent, often limited to factual teaching, with variable coverage across medicine, pharmacy, nursing, dentistry, veterinary medicine and midwifery. Applying a behavioural science framework such as COM-B enables evaluation of whether interventions target the capability, opportunity and motivation influences that underpin stewardship practice.

**Objectives:**

This review aimed to (i) identify AMS educational interventions delivered to undergraduate and pre-registration healthcare students across disciplines, and (ii) evaluate the extent to which they addressed behavioural coverage across COM-B influences necessary to prepare students for stewardship practice.

**Methods:**

A protocol was registered on PROSPERO (CRD420250655653). Six databases (PubMed, Web of Science, PsycINFO, EMBASE, CINAHL Plus, Scopus) were searched on 4 February 2025. Peer-reviewed studies in English evaluating AMS education for pre-registration students in medicine, pharmacy, nursing, dentistry, veterinary medicine and midwifery were included. Eligible interventions occurred in university or supervised clinical settings and reported at least one learning outcome relevant to stewardship. Data extraction was conducted in duplicate, with quality assessed using an adapted Medical Education Research Study Quality Instrument (MERSQI). A narrative synthesis was undertaken, coding interventions against COM-B domains adapted to an educational context.

**Results:**

Of 7771 records screened, 42 studies were included, involving 8567 students across six continents. Mot used pre/post designs; two were randomized controlled trials. All addressed psychological capability (42/42), typically through lectures, online modules, or case-based reasoning tasks. Reflective motivation was supported in 25/42, often by strengthening prescribing confidence or professional identity. Fewer interventions created physical opportunity (20/42), such as access to prescribing charts, susceptibility data, or digital tools, or social opportunity (18/42) through teamwork and supervision. Only 9/42 developed physical capability via supervised practice or simulation. Just 2/42 addressed automatic motivation, such as the stress of time-pressured escape rooms or the satisfaction from user-friendly prescribing apps, showing how emotions can shape whether and how stewardship behaviours are carried out. Overall methodological quality was moderate (MERSQI mean=10/18).

**Conclusions:**

Undergraduate AMS education is widespread but uneven in its behavioural coverage across COM-B influences. Interventions predominantly focus on knowledge, with less attention to the skills, opportunities and motivational influences that enable stewardship in practice. COM-B highlights the need for curricula that combine factual teaching with rehearsal of practical skills, authentic resources, collaborative teamwork, reflective role identity and positive engagement. Embedding behavioural science principles into AMS education will better equip graduates to meet the complex stewardship demands of future healthcare.

